# Supplementary material for: Soluble SIGLEC5: A New Prognosis Marker in Colorectal Cancer Patients
Source: Cancers (Basel). 2021 Aug 2;13(15):3896. doi: 10.3390/cancers13153896 (PMC8345516; doi:10.3390/cancers13153896)
Supplement: Supplementary file 1 [file cancers-13-03896-s001.zip › cancers-1297765-supplementary.pdf]

cancers

MDPI

Supplementary

Soluble SIGLEC5: A New Prognosis Marker in Colorectal Cancer Patients

Karla Montalbán-Hernández et al.

Table S1. Predictive values for the different survival factors.

Survival Factor	AUC	95% CI	Sensitivity	Specificity	PPV	NPV	Chi-squared	*p* value
sSIGLEC5	0.853	0.7729–0.9344	1	0.67	0.26	1	19.15	*<0.0001**** *
TNM	0.715	0.5754–0.8546	1	0.23	0.13	1	3.77	*0.0522*
Therapeutic Information	0.5207	0.3294–0.7119	0.45	0.54	0.1	0.9	0.088	*0.0881*

****, $p < 0.0001$

Table S2. Eliminated variables from logistic regression model.

		Score	*p* value
**Variables**	Age	1,164	0,281
	Sex	0,817	0,366
	Disease Stage	0,198	0,656
	Perineural Invasion	1,080	0,299
	Lymphovascular Invasion	1,439	0,230
	Chemotherapy	0,067	0,796
	TNM – Tumour Size	1,151	0,283
	TNM – Node Infiltration	3,050	0,081
	TNM - Metastasis	2,217	0,136
	Surgical Margins	0,001	0,978
	CEA	2,187	0,139
**Global Statistics**		14,450	0,209

Wald forward conditional stepwise regression, including age, gender, disease stage, treatment, tumour size, metastasis, node infiltration, lymphovascular invasion, perineural invasion, surgical margins, CEA, tumour dedifferentiation and sSIGLEC5 levels as variables. Eliminated variables after two steps are shown. Units: age in years and CEA in ng/mL.

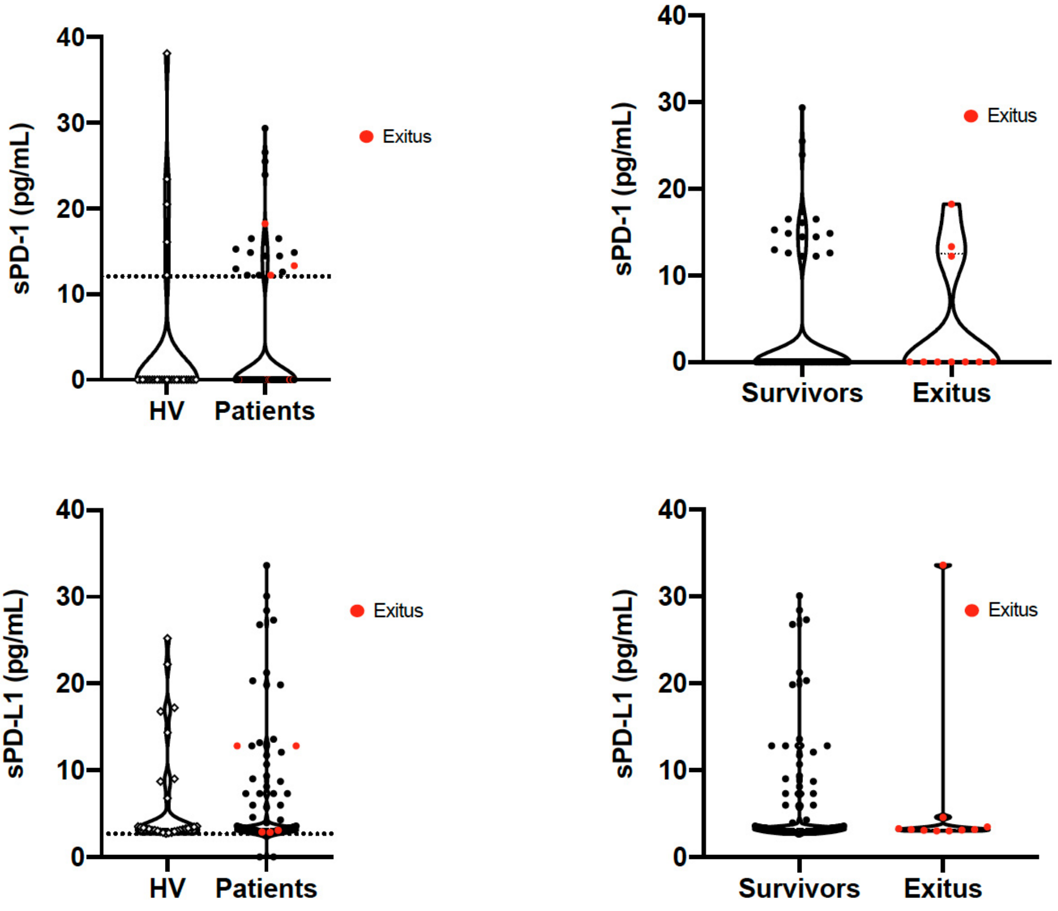

Figure S1. Pre-operative soluble immune checkpoints in colorectal cancer patients.

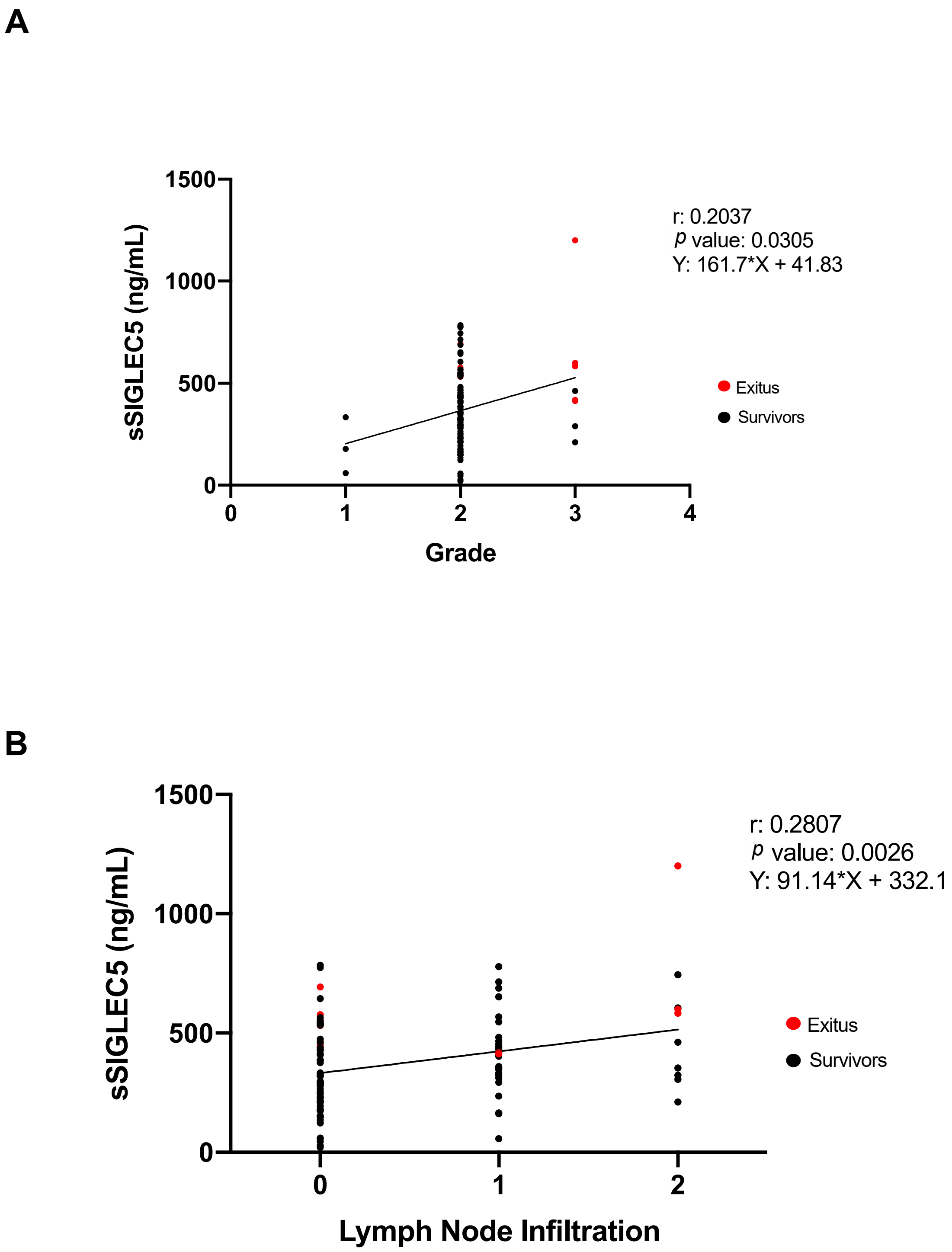

Figure S2. Pre-operative sSIGLEC5 levels correlate with (A) tumour dedifferentiation and (B) lymph node metastasis, and Table S1: Predictive values for the different survival factors.
